# Multiple sclerosis is linked to MAPK^ERK^ overactivity in microglia

**DOI:** 10.1007/s00109-021-02080-4

**Published:** 2021-05-05

**Authors:** George J. A. ten Bosch, Jolande Bolk, Bert A. ‘t Hart, Jon D. Laman

**Affiliations:** 1 Department of Medical Oncology, Leiden University Medical Center, P.O. Box 9600, 2300 RC Leiden, The Netherlands; 2 Department of Anesthesiology, Medisch Spectrum Twente, Enschede, The Netherlands; 3 Department Anatomy and Neuroscience, Amsterdam University Medical Center (VUmc), Amsterdam, The Netherlands; 4 Department Biomedical Sciences of Cells & Systems, University Medical Center Groningen, Groningen, The Netherlands

**Keywords:** MAPK^ERK^; Multiple sclerosis, DUSP6, LMP-1, Microglia, Demyelination

## Abstract

Reassessment of published observations in patients with multiple sclerosis (MS) suggests a microglial malfunction due to inappropriate (over)activity of the mitogen-activated protein kinase pathway ERK (MAPK^ERK^). These observations regard biochemistry as well as epigenetics, and all indicate involvement of this pathway. Recent preclinical research on neurodegeneration already pointed towards a role of MAPK pathways, in particular MAPK^ERK^. This is important as microglia with overactive MAPK have been identified to disturb local oligodendrocytes which can lead to locoregional demyelination, hallmark of MS. This constitutes a new concept on pathophysiology of MS, besides the prevailing view, i.e., autoimmunity. Acknowledged risk factors for MS, such as EBV infection, hypovitaminosis D, and smoking, all downregulate MAPK^ERK^ negative feedback phosphatases that normally regulate MAPK^ERK^ activity. Consequently, these factors may contribute to inappropriate MAPK^ERK^ overactivity, and thereby to neurodegeneration. Also, MAPK^ERK^ overactivity in microglia, as a factor in the pathophysiology of MS, could explain ongoing neurodegeneration in MS patients despite optimized immunosuppressive or immunomodulatory treatment. Currently, for these patients with progressive disease, no effective treatment exists. In such refractory MS, targeting the cause of overactive MAPK^ERK^ in microglia merits further investigation as this phenomenon may imply a novel treatment approach.

## Introduction

More than 160 years after Jean-Martin Charcot’s description of MS, the pathophysiology of this neurodegenerative disease is still rather enigmatic. The paradigm most adhered to is that MS is caused by autoimmune reactivity against central nervous system (CNS)-antigens. This concept is supported by clinical benefits of immune suppression and immunomodulation, and these approaches represent the mainstay of contemporary MS treatment. However, this concept does not explain why many patients eventually deteriorate neurologically despite optimized immunomodulation. Such condition is common in patients with progressive MS, but often this befalls also MS patients that initially responded favorably to immunosuppression but eventually become refractory to this approach. As this shortcoming of treatment of refractory MS constitutes a remarkable dichotomy in the disease (effectively treatable MS versus refractory MS), this contrast propels the quest for further understanding the mechanisms behind the disease and, by this, the search for effective treatment with regard to this pathophysiology. In this review, literature that points to overactivity of mitogen-activated protein kinases (MAPK) in MS, in particular MAPK^ERK^, is summarized. It appears that overactivity of MAPK^ERK^ in MS microglia can lead to locoregional inflammation within the CNS besides dysfunction of regional oligodendrocytes.

In view of the overall pathogenic complexity, several mechanisms have been considered to contribute to this devastating neurodegenerative disease. The interpretation of data on MS presented here is novel and signifies another mechanism that can explain and unify phenotypic characteristics of MS.

## Preclinical indications on involvement of MAPK in neurodegeneration

### TAK1 in microglia

**Fig. 1:**
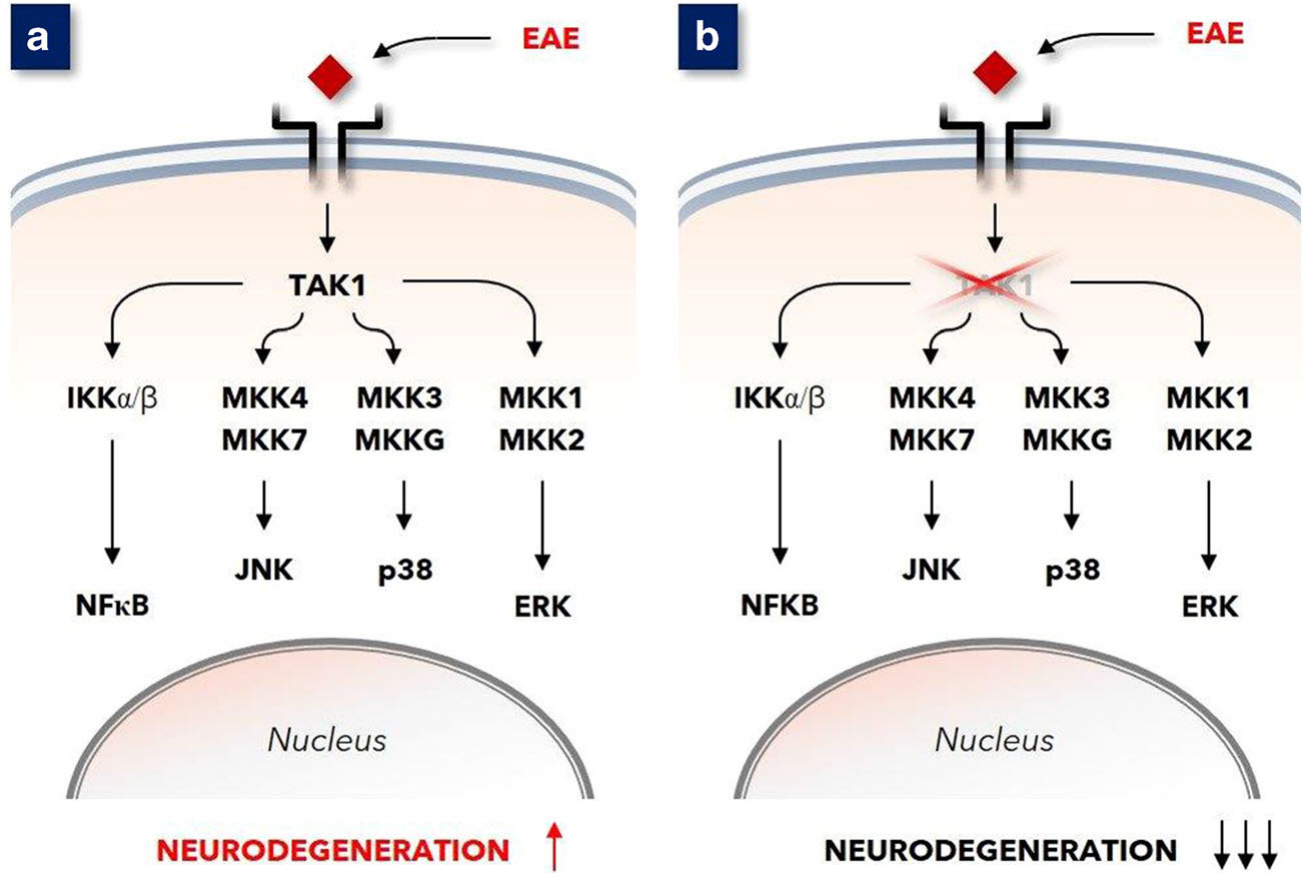
**a** In mice immunized with MOG35–55 peptide, the expression of TAK1 in microglia appeared essential for the development of autoimmune inflammation of the CNS. **b** Mice with TAK1-deficient microglia were highly resistant to MOG35–55 immunization, which resulted in a considerably less severe disease [1]

In 2013 Goldmann and co-workers demonstrated that microglia-endogenous TGFβ-activated kinase-1 (TAK1) is a key component in the regulation of CNS inflammation [1]. In mice with induced experimental autoimmune encephalopathy (EAE, a model for MS), depleting TAK1 in microglia ameliorated clinical disease manifestations, reduced CNS inflammation, and diminished axonal and myelin damage (see Fig. 1a and b). Depleted TAK1 function in microglia inhibited NF-κB and signaling via the mitogen-activated protein kinase (MAPK) pathways JNK, p38, and ERK (MAPK^JNK^, MAPK^p38^, and MAPK^ERK^, respectively). In the context of the pathophysiology of MS, these observations draw attention to these very pathways. This in particular since activation of these pathways in microglia induces cytokine release into the microenvironment that can lead to local inflammation in the CNS [2–4]. This work by Goldmann et al. focused on a role of MAPK pathways in a murine model of MS, EAE.

### BRAF^V600E^ in microglia

Recently, Mass and co-workers showed that the induction of MAPK^ERK^ pathway overactivity in mouse microglial cells resulted in neurodegeneration [5]. Expression of BRAF^V600E^, a mutated form of the gene *BRAF*, which encodes the oncogenic B-Raf kinase, leads to substantial overactivity of the MAPK^ERK^ pathway, a phenomenon widely acknowledged in today’s clinical oncology and hemato-oncology practice [6]. Mass et al. investigated neurodegeneration that occurs in the context of neurohistiocytosis, as this disease can harbor BRAF^V600E^ [7].

**Fig. 2:**
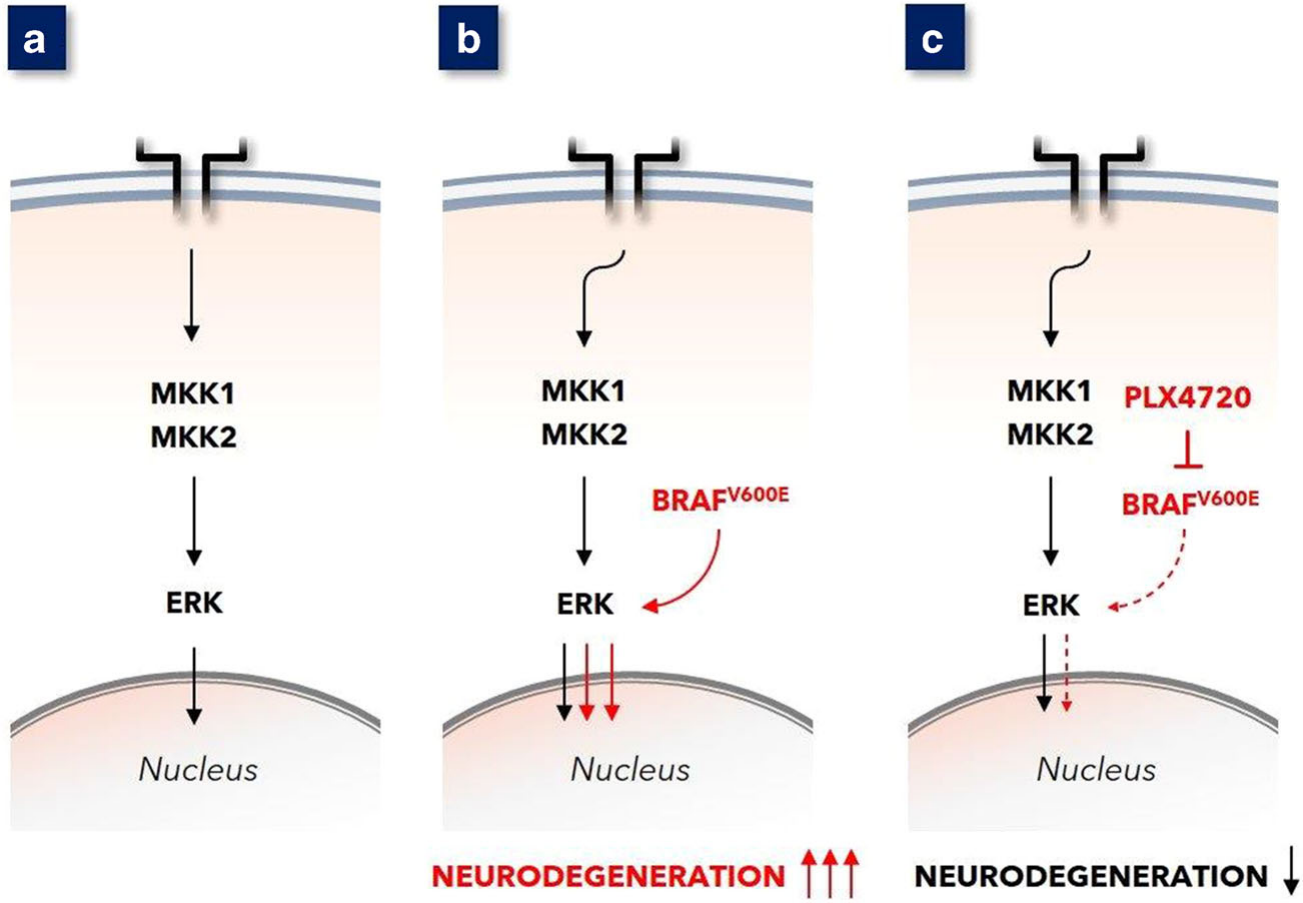
**a **Ligand binding to surface receptor (e.g. EGF-R) evokes downstream signaling leading to MAPK^ERK^ activation. **b** The introduction of BRAF^V600E^ in mouse microglia leads to substantial overactivation of MAPK^ERK^. This resulted in clinical and histopathological substantial neurodegeneration. **c** Early administration of BRAF^V600E^-inhibiting PLX4720 to these mice with BRAF^V600E^ expressing microglia gave diminished MAPK^ERK^ activation in these microglia and clinically as well as histopathologically attenuated neurodegeneration [5]

Mass et al. introduced this BRAF^V600E^ expression in erythro-myeloid progenitor cells that give rise to microglia, the tissue-resident macrophages of the CNS. The ensuing modification of MAPK^ERK^ overexpression in mouse microglia cells within the CNS resulted in late-onset neurodegeneration (see Fig. 2a and b) with progressive hindlimb paresis. Notably, the very induction of MAPK^ERK^ overexpression in murine microglia also resulted in clinical and histopathological deviations in vivo: amoeboid microglia, GFAP-positive astrogliosis, expression of the PDGFα receptor as well as VLA4 and CD11a, deposition of amyloid precursor protein, and synaptic loss. Phenomena included localized demyelination and, finally, neural death. These histopathological features are identical to those found in human MS lesions. Importantly, Mass et al. observed that treatment of mice with BRAF^V600E^-overexpressing microglia with the BRAF^V600E^inhibitor PLX4720 mitigated disease progression as well as prevented histopathological aberrations (see Fig. 2c).

The observations in mice illustrate that inducing MAPK^ERK^ pathway overactivity leads to late-onset progressive paresis and histopathology that resembles MS. These findings gain even more weight by reciprocity: the demonstration that blocking of MAPK^ERK^ pathway overactivation, by BRAF^V600E^ inhibition, mitigated the progression of neurodegeneration.

Combination of the reports by Goldmann [1] and Mass [5], both based on a *mouse model* of neurodegeneration, with the abundant data on biochemical and epigenetic signals in MS *in man* (refer to Tables 1 and 2), points to a likely pivotal role of MAPK^ERK^ pathway overactivity in microglia in the neurodegenerative pathology of MS.

**Table 1:** Examples of biochemical associations between MS and the MAPK^ERK^ pathway

Parameter	Association	References
Wnt/β-catenin	Reduction in Wnt/β-catenin signaling in microglia leads to a microglial phenotype causing hypomyelination This Wnt/β-catenin signaling is downregulated by overactivity of the MAPK^ERK^ pathway	[8, 9]
MSK1	The mitogen- and stress-activated kinase 1 (MSK1) phosphorylates pro-inflammatory nuclear factor NF-κB p65. MSK1 is activated by MAPK^ERK^ and MAPK^p38^ MS-medicine dimethyl fumarate (Tecfidera®) is known to inhibit MSK1 besides counteracting oxidative stress, also in microglia	[10–12]
MC1r	The melanocortin 1 receptor (MC1r), also expressed on microglia, is involved in signal transduction and development. In comparison with default ligand α-MSH, [Nle^4^, DPhe^7^]-α-MSH leads to inhibition of phosphorylation of ERK This [Nle^4^, DPhe^7^]-α-MSH appeared neuroprotective in murine models of neuroinflammation	[13, 14]
Notch1	Activation of Notch1 by ligands Jagged1 or contactin are associated with decreased oligodendrocyte precursor cells and demyelination in MS Expression of these ligands seems linked to MAPK^ERK^ induced TGFβ (leads to Jagged1), and to MAPK^ERK^ activity–dependent contactin1, respectively	[15–18] [18, 19]
MITF	Myelin basic protein (MBP) gene expression appears regulated by microphthalmia-associated transcription factor (MITF) Sustained ERK phosphorylation stimulates degradation of MITF, thus overactive MAPK^ERK^ may hinder expression of MBP	[19, 20]
DHODH	Teriflunomide (Aubagio®, drug registered for MS) inhibits dihydro-orotate dehydrogenase (DHODH), a key enzyme in the pyrimidine synthesis pathway DHODH is regulated at the level of carbamoyl-phosphate synthetase(CAD), an enzyme activated by MAPK^ERK^ phosphorylation Therefore, as cytokine production is dependent on DHODH-directed pyrimidine synthesis and the functioning of CAD/DHODH is lowered by teriflunomide in microglia, this may point to activity of MAPK^ERK^ in MS	[21–23]
VCAM-1	Inhibition of the MAPK^ERK^ pathway downregulates the expression of vascular cell adhesion molecule 1 (VCAM-1), ligand for integrin α4β1. As a key adhesion molecule integrin α4β1 induces the translocation of leukocytes to inflamed tissue. This demonstrates a role of the MAPK^ERK^ in activating this integrin, also in microglia Therefore, controlling overactivity in the MAPK^ERK^ pathway may result in a similar limitation of integrin α4β1 activation as applying by α4β1-antagonist MoAb natalizumab (Tysabri®, medicine for MS)	[24–26]
NfL	Activation of MAPK^ERK^ (and also MAPK^p38^) leads to expression of Neurofilament light (NfL) protein As the expression level of NfL is positively associated with the level of MS disease activity (relapse rate, Expanded Disability Status Scale score, Age-Related MS Severity Score, and MS Impact Score) the activity of MAPK^ERK^ (and also MAPK^p38^) relates to MS	[27, 28]
GFAP	GFAP (Glial Fibrillary Acidic Protein) is known to participate in glial scarring as a consequence of neurodegenerative conditions. It is an established biomarker of neurodegeneration in MS, besides NfL In 2013 it was observed that preventing MAPK^ERK^ activation antagonized IL-1β-induced GFAP expression, whereas overactive MAPK^ERK^ appeared to contribute to expression of GFAP	[29–32]
MMP-9	MMP-9 (matrix metalloproteinase 9) is involved in blood-brain barrier disruption and formation of MS lesions. In patients with MS, the expression of MMP-9 is substantially higher when compared with controls, and it can be considered biomarker for disease severity MMP-9 expression occurs in response to activation of the MAPK^ERK^ pathway	[33–35]

**Table 2:** Similarities between MS and MAPK^ERK^ pathway associated microRNA

Parameter	Association	References
miRNA-21	MicroRNA-21 is upregulated in CSF, and also found in brain white matter lesions in patients with MS Sprouty2 (SPRY2), as a critical negative regulator of MAPK^ERK^ signaling, is a target of miRNA-21. Consequently, MAPK^ERK^ signaling pathway activation is upregulated as a consequence of SPRY2 due to higher expression of this microRNA	[36–38]
miRNA-30d	miR-30d is found enriched in feces of patients with untreated MS. Synthetic miR-30d given orally ameliorates the effects of experimental autoimmune encephalomyelitis (EAE, model of MS) in mice miRNA-30d is identified to suppress the MEK/ERK and PI3K/Akt pathways, and this supports a role of MAPK^ERK^ in MS	[39, 40]
miRNA-101	MicroRNA-101 participates in the regulation of MAPKs as it targets MAPK Phosphatase-1 (MKP-1). As negative feedback control enzyme system, MKP-1 also dephosphorylates MAPK^ERK^ besides MAPK^p38^ In patients with MS miRNA-101 has been identified, in particular in those with RRMS	[41–44]
miRNA-145	Dual-specificity phosphatase 6 (DUSP6, or MKP3) is a cytoplasmic phosphatase with high specificity for MAPK^ERK^ extracellular signal-regulated kinase (ERK) miRNA-145 is identified to target directly DUSP6 The miR-145 appears up-regulated in MS, in PBMC as well as in MS lesions p53 expression is higher in MS lesions, and this p53 can lead to miR-145 upregulation. By this, DUSP6 can be targeted which leads to lower negative feedback on MAPK^ERK^	[45–49] [50] [50, 51] [48, 49, 52] [52]
miRNA-146a	Analysis of miRNA in CSF and active lesions in patients with MS show upregulation of miR-146a and miR-146b Transcription of miR-146a and miR-146b appears upregulated via different MAP kinase pathways. miR-146b expression is MAPK^JNK1/2^ and MAPK^ERK^ dependent miRNA-146a is upregulated by the EBV encoded protein LMP-1. Both are linked to MAPK^ERK^ activity	[37, 51] [53, 54]
miRNA-219	In chronic MS lesions miR-219 is found *down*regulated In GBM samples miRNA-219-5p was found to inhibit RAS-MAPK and PI3K pathways	[55, 56]
miRNA-221	miR-221-3p is found in higher levels in blood of MS patients. Its expression may relate to neurogenesis in the context of neural regulation The MAPK^ERK^ activity was found to promote an increase in miR-221	[57, 58]
miRNA-338	miRNA-338 is downregulated in chronic MS lesions This miRNA inhibits the MAPK^ERK^-signaling pathway: when *over*expressed in GBM a lower expression of MEK-2 and ERK-1 was observed	[55] [59]
miRNA-564	In patients with MS, miRNA-564 has been identified to be downregulated in T-cells (whether any level of this miRNA is lymphocytogenic or whether it originates from intercellular exchange is not analyzed) miRNA-564 has been identified to target pERK	[60–62]

## Association of MAPK overactivity with MS

Separate observations on several biochemical phenomena in MS hint at the involvement of, in particular, the MAPK^ERK^ pathway. These observations include the Wnt/β-catenin pathway, sphingosine 1-phosphate (S1P), mitogen- and stress-activated kinase-1 (MSK1), melanocortin 1 receptor (MC1r), microphthalmia-associated transcription factor (MITF), carbamoyl-phosphate synthetase (CAD), and vascular cell adhesion molecule 1 (VCAM1). All are mechanistically linked to MAPK^ERK^ as well as to MS. These and several other associations between MS and the MAPK^ERK^ pathway are described concisely in Table 1. The attenuation of MS disease after inhibition of the MAPK^ERK^ pathway and associated factors can be considered to affirm the involvement of this pathway in MS.

Besides, certain microRNAs (miRNA) have been detected in deviant expression levels in patients with MS, when compared to non-MS individuals. These miRNAs play a role in controlling of—or responding to—the activation status of the MAPK^ERK^ pathway (Table 2). The relationship of these MS-associated miRNAs with MAPK^ERK^ activity also suggests a particular relevance of MAPK^ERK^ activity in MS.

In short, the similarities between the role of MAPK activity in microglia in causing mouse neurodegeneration and observations in human disease MS pinpoint overactive MAPK^ERK^ as primary involved process. In line, the occurrence of MS-associated miRNAs appears associated with MAPK^ERK^ activity.

## From MAPK^ERK^ to demyelination, hallmark of MS

There is a direct causal relation between clinical manifestations in MS with the pathological hallmarks (inflammation, neurodegeneration, and demyelination). Therefore, when considering MAPK^ERK^ overactivity in microglia as a *common thread* in MS, a negative influence by these affected microglia on oligodendrocytes regarding myelination substantiates linkage between these cell types. In fact, such crosstalk between microglia and adjacent oligodendrocytes has been reported in 2014 by Peferoen and colleagues [3]. Earlier, it was found that affected microglia have a detrimental effect on adjacent oligodendrocytes [63]. In line, oligodendrocytes appeared particularly susceptible to microglia-emitted factors in reaction to MAPK^ERK^ activity [64]. Microglial MAPK^ERK^-induced cytokines include IL-1β and TNF-α [65, 66], and these cytokines damage locoregional oligodendrocytes resulting in hypomyelination. Taken the essential role of oligodendrocytes in the trophic support of axons any insult to oligodendrocytes will also impact on axonal physiology [67, 68].

Together, microglia can influence oligodendrocytes in an unfavorable fashion, and this can explain local demyelination in response to regional MAPK^ERK^-overexpressing microglia. Moreover, MAPK^ERK^ activation in microglia has been identified to lead to emission of various pro-inflammatory mediators [69] further contributing to sclerosis of affected tissue within the CNS.

## On MS phenotypes

The individual MS patient’s disease status is usually described as one of four recognized phenotypes [70]. These phenotypes, or actual clinical course descriptions, are designated clinically isolated syndrome (CIS), relapsing remitting MS (RRMS), secondary progressive MS (SPMS), and primary progressive MS (PPMS). All phenotypes are defined by several parameters including disease history, actual relapse rate, and disease progression status [71].

One peculiar distinction between these MS phenotypes is the varying benefit from anti-inflammatory and immunomodulatory treatment. While clinically relevant responses to these therapies can be observed in RRMS, in progressive MS phenotypes and/or when there is a longer period of time after diagnosis, these treatment modalities show modest or no beneficial effect anymore. This is a meaningful distinction as it implies that MS neurodegeneration is most probably caused by other factors than influenceable inflammation alone.

It was proposed that the different MS phenotypes may be variations on a central theme [72]. Conceptually, symptoms and pathology of progressive MS are caused by neurodegeneration leading to dysfunctional axon-myelin units, while relapses are due to immune hyper-reactivity against antigens released from degenerating units. Histopathological evidence of MS pathology preceding autoimmune neuropathology includes dysfunctional axon-myelin units [73] and nodules of reactive microglia surrounding degenerating axons [74].

It may thus well be that the neuroinflammation is an effect that is superimposed on or occurs in parallel to the consequences of overactive MAPK^ERK^ in microglia. Both mechanisms can be explained by microglia-endogenous enzymes downstream TAK1 [1]. Disturbances of downstream TAK1 can lead to both overactive MAPK^ERK^ and overactive MAPK^p38^ [75]. Activated MAPK^p38^ is classically associated with inflammation [50, 51], and in the CNS, this may cause damage separate from the neurodegeneration caused by overactive MAPK^ERK^. Such mechanistic diversity in pathogenesis could explain the divergent clinical courses known from MS disease phenotypes [70].

## Causes of MAPK overactivity

Overactivation of MAPK pathways can be the result of several different mechanisms. Besides activation as result of extracellular receptor tyrosine kinase (RTK)-ligand binding, also intracellular processes can lead to overactivity of this pathway. These include activating mutations in genes encoding proteins that constitute this pathway but with elevated kinase activity. BRAF^V600E^ is one example of such gene mutation-derived protein that leads to substantially higher kinase activity. BRAF^V600E^ is well known for its role in several neoplasms [6]. To date such mutations have not been detected in MS.

MAPK signaling in the cell is controlled by negative feedback systems consisting of dedicated phosphatases (dual-specificity phosphatases (DUSP), also called mitogen-activated protein kinase phosphatases (MKP)). Therefore, an alternative explanation for an overactivated MAPK signaling is failure of this feedback regulation. When these MAPK controlling negative feedback systems fail, MAPK pathway phosphorylating activity becomes uncontrolled, and this results in inadequate higher kinase activity [76]. For instance, miRNA-145 is highly expressed in MS tissue [77, 78]. This miRNA-145 can downregulate DUSP6 [79], and this results in an overactivation of MAPK^ERK^. Moreover, this overactivation of MAPK^ERK^ in microglia can also be the consequence of other factors related to MS, for instance infection with neurotropic viruses like Epstein Barr virus (EBV). Such infection can result in the pathogenic disturbance of intracellular biochemistry leading to overactivation of MAPK^ERK^.

## Possible associations of MS risk factors with MAPK^ERK^ overactivity

Broadly acknowledged risk factors for MS development and progression are low serum vitamin D levels at diagnosis, tobacco smoking, and prior infection with EBV. A common denominator of these factors is that they all negatively affect specific dual-specificity MAP kinase phosphatases (DUSPs). As these MS risk factors all downregulate DUSPs, this could explain MAPK overactivity.

### Hypovitaminosis D

Vitamin D supplementation leads to higher DUSP1 levels [80]. As DUSP1 counteracts overactivity of MAPK^p38^, and to a lesser extent also of MAPK^ERK^ [80–84], a *shortage* of vitamin D might result in higher activity of MAPKs, and in particular of MAPK^p38^. Conversely, as adequate levels of vitamin D facilitate regulation of DUSP1, this can contribute to better controlled MAPK^p38^ activity and thereby to reduction of MAPK-induced inflammation [85]. Importantly, vitamin D supplementation has been found to consolidate or improve the clinical condition of patients with MS *only early* after disease onset [86], and this may illustrate that MAPK^p38^-related inflammation is clinically relevant primarily in *early phases* of MS. Refractoriness to immunosuppressive/immunomodulatory measures, observed often in longer existing progressive phenotypes of MS, also reflects MAPK^p38^ inflammation-independent neurodegeneration in later stages of the disease, as in these patients the neurodegeneration in the context of MS invariably proceeds. The MAPK^p38^ interference in early stages of MS demonstrates clinical relevance of vitamin D suppletion. In short, hypovitaminosis D seems to diminish activity of DUSP1 in MS, while supplementation of vitamin D shows clinically benefit solely in early stages of MS. These data suggest that in longer existing, progressive stages of MS, other processes are involved that lead to ongoing neurodegeneration. Consequently, in view of the here discussed role of MARK^ERK^ in MS, also other MAPK phosphatases must be involved, in particular in longer existing or progressive phenotypes of MS, as here classical immune suppression/immune modulation shows infective.

### EBV infection

**Fig. 3:**
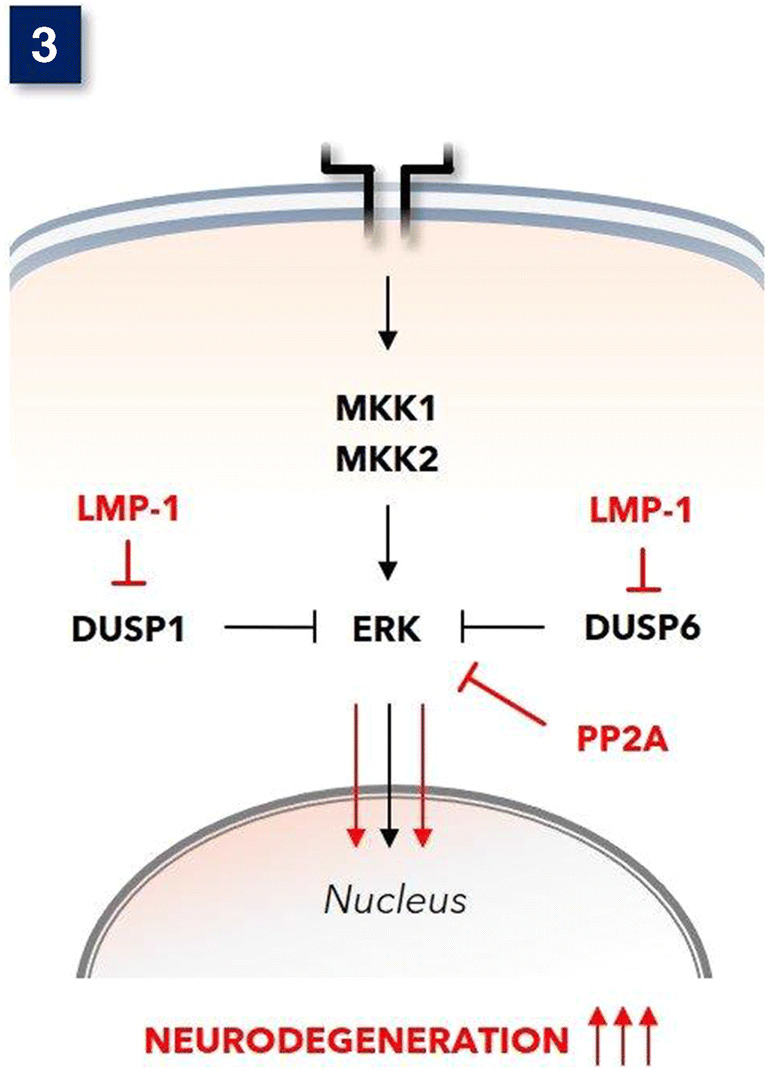
Under physiological circumstances, the MAPK^ERK^ is adequately controlled by negative feedback phosphatases, in particular DUSP-6 and also DUSP-1. Epstein Barr virus (EBV)-encoded Latent Membrane Protein-1 (LMP-1) represses these phosphatases in cells with EBV latency [89]. Fingolimod activates protein serine/threonine phosphatase 2A (PP2A) [91], and this can dephosphorylate MAPK^ERK^ [92]

Virus infection, in particular infection with Epstein Barr virus (EBV), causes MAPK^ERK^ overactivity [87], which is probably mediated by downregulation of DUSP6 (MKP-3) and DUSP-8. EBV proteins, including Epstein Barr nuclear antigen-2 (EBNA2) [88] and latent membrane protein-1 (LMP-1) [87], are the most straightforward cause for the MAPK^ERK^ upregulating properties of EBV. Indeed, EBV infection of microglia can eventually lead to virus latency [89], and the latency-encoded LMP-1 has been proven to substantially downregulate DUSP6 and also DUSP1 [90] resulting in overactivation of MAPK^ERK^ (see Fig. 3). Furthermore, infection with EBV is presumably needed for reactivation of human endogenous retrovirus (HERV) group K(HML2), to which belongs the in MS encountered HERV-K18 [93]. This HERV-K18 by itself also participates in MAPK^ERK^ pathway activation effectively [94].

In general, these acknowledged risk factors for MS development and progression can all be related to MAPK induced inflammation. The disappointing efficacy of immunosuppression/immunomodulation in progressive MS may imply that neurodegeneration here is driven by other mechanisms than those sensitive to present day anti-MS medicines that are effective often only temporarily and in earlier stages of the disease.

## Conclusions and future directions

A prominent early pathological feature of MS, that precedes the autoimmune attack, is the presence of microglia nodules, which are composed of activated microglia centering on a degenerating axon [74]. Although the exact induction mechanism of the nodules is not known, the expression of IL-1β indicates MAPK pathway activation [95]. It has been proposed that a proportion of these nodules stimulate the development of inflammatory pathology that is the pathological hallmark of MS [96]. This publication provides a possible explanation for this early aberrant behavior of microglia that disturbs its normal homeostatic functions.

Reassessment of published data reveals that overactivation of MAPK, in particular MAPK^ERK^, in microglia is unambiguously linked to MS. We posit that as this mechanism is different from other pro-inflammatory stimuli in microglia (e.g., MAPK^p38^ activation), it can explain that MS patients with progressive phenotypes experience ongoing neurodegeneration despite adequate immunosuppression or immunomodulation. Of note, immunosuppression and immunomodulation do affect pro-inflammatory effects of MAPK^p38^, but these do not affect the mechanisms of MAPK^ERK^ overactivation in microglia. Consequently, neutralization of MAPK^ERK^ overactivity in microglia may be a feasible approach to halt the negative effect of affected microglia on oligodendrocytes and by this achieve attenuation of MS-associated demyelination. The observation that established risk factors for MS all have been found to downregulate MAPK activity-controlling phosphatases (DUSPs), a possible pathogenic role of MAPKs, in particular the observed MAPK^ERK^ overactivity, is emphasized.

In view of the fact that the MAPK families constitute indispensable and crucial enzymatic pathways for every cell, inhibition of the MAPK^ERK^ pathway is potentially detrimental. This is illustrated by the vast repertoire of severe adverse events observed after the systemic application of anti-neoplastic medicines developed for the inhibition of MAPK^ERK^ overactive disease (e.g., inhibitors of BRAF^V600E^, MEK, or KRAS^G12C^).

Neutralization of MAPK^ERK^ overactivity in MS may be achieved by correcting the activity of DUSPs that can be responsible for insufficient negative feedback of the MAP kinases involved. As MAPK^ERK^ overactivity has been found an effect of the EBV latency-encoded LMP-1 [87], this viral protein should be neutralized in order to abrogate the pathological process with seems current in MS. Indeed, higher expression of this LMP-1 has been observed in the brain of patients with MS [94]. Therefore, LMP-1-targeted siRNA [97], RNAi, or possibly LMP-1-directed CRISPR-cas9 [98] may constitute treatment modalities for MS. Finally, patients with MS that show refractory for any contemporary treatment, for instance those suffering from long term progressive phenotypes, could benefit from such approach, as these patients suffer from neurodegeneration unabatedly.

## References

[1] Goldmann T, Wieghofer P, Muller PF, Wolf Y, Varol D, Yona S, Brendecke SM, Kierdorf K, Staszewski O, Datta M (2013). A new type of microglia gene targeting shows TAK1 to be pivotal in CNS autoimmune inflammation. Nat Neurosci.

[2] Hanisch UK (2002). Microglia as a source and target of cytokines. Glia.

[3] Peferoen L, Kipp M, van der Valk P, van Noort JM, Amor S (2014). Oligodendrocyte-microglia cross-talk in the central nervous system. Immunology.

[4] Bachstetter AD, Van Eldik LJ (2010). The p38 MAP kinase family as regulators of proinflammatory cytokine production in degenerative diseases of the CNS. Aging Dis.

[5] Mass E, Jacome-Galarza CE, Blank T, Lazarov T, Durham BH, Ozkaya N, Pastore A, Schwabenland M, Chung YR, Rosenblum MK, Prinz M, Abdel-Wahab O, Geissmann F (2017). A somatic mutation in erythro-myeloid progenitors causes neurodegenerative disease. Nature.

[6] Davies H, Bignell GR, Cox C, Stephens P, Edkins S, Clegg S, Teague J, Woffendin H, Garnett MJ, Bottomley W, Davis N, Dicks E, Ewing R, Floyd Y, Gray K, Hall S, Hawes R, Hughes J, Kosmidou V, Menzies A, Mould C, Parker A, Stevens C, Watt S, Hooper S, Wilson R, Jayatilake H, Gusterson BA, Cooper C, Shipley J, Hargrave D, Pritchard-Jones K, Maitland N, Chenevix-Trench G, Riggins GJ, Bigner DD, Palmieri G, Cossu A, Flanagan A, Nicholson A, Ho JWC, Leung SY, Yuen ST, Weber BL, Seigler HF, Darrow TL, Paterson H, Marais R, Marshall CJ, Wooster R, Stratton MR, Futreal PA (2002). Mutations of the BRAF gene in human cancer. Nature.

[7] Thacker NH, Abla O (2019). Pediatric Langerhans cell histiocytosis: state of the science and future directions. Clin Adv Hematol Oncol.

[8] Van Steenwinckel J, Schang AL, Krishnan ML, Degos V, Delahaye-Duriez A, Bokobza C, Csaba Z, Verdonk F, Montane A, Sigaut S (2019). Decreased microglial Wnt/beta-catenin signalling drives microglial pro-inflammatory activation in the developing brain. Brain.

[9] Biechele TL, Kulikauskas RM, Toroni RA, Lucero OM, Swift RD, James RG, Robin NC, Dawson DW, Moon RT, Chien AJ (2012) Wnt/beta-catenin signaling and AXIN1 regulate apoptosis triggered by inhibition of the mutant kinase BRAFV600E in human melanoma. Sci Signal 5: ra3. 10.1126/scisignal.2002274 10.1126/scisignal.2002274 PMC3297477 22234612

[10] Yue Y, Stone S, Lin W (2018). Role of nuclear factor kappaB in multiple sclerosis and experimental autoimmune encephalomyelitis. Neural Regen Res.

[11] Peng H, Guerau-de-Arellano M, Mehta VB, Yang Y, Huss DJ, Papenfuss TL, Lovett-Racke AE, Racke MK (2012). Dimethyl fumarate inhibits dendritic cell maturation via nuclear factor kappaB (NF-kappaB) and extracellular signal-regulated kinase 1 and 2 (ERK1/2) and mitogen stress-activated kinase 1 (MSK1) signaling. J Biol Chem.

[12] Paraiso HC, Kuo PC, Curfman ET, Moon HJ, Sweazey RD, Yen JH, Chang FL, Yu IC (2018). Dimethyl fumarate attenuates reactive microglia and long-term memory deficits following systemic immune challenge. J Neuroinflammation.

[13] Rennalls LP, Seidl T, Larkin JM, Wellbrock C, Gore ME, Eisen T, Bruno L (2010). The melanocortin receptor agonist NDP-MSH impairs the allostimulatory function of dendritic cells. Immunology.

[14] Mykicki N, Herrmann AM, Schwab N, Deenen R, Sparwasser T, Limmer A, Wachsmuth L, Klotz L, Kohrer K, Faber C et al (2016) Melanocortin-1 receptor activation is neuroprotective in mouse models of neuroinflammatory disease. Sci Transl Med 8: 362ra146. 10.1126/scitranslmed.aaf8732 10.1126/scitranslmed.aaf8732 27797962

[15] John GR, Shankar SL, Shafit-Zagardo B, Massimi A, Lee SC, Raine CS, Brosnan CF (2002). Multiple sclerosis: re-expression of a developmental pathway that restricts oligodendrocyte maturation. Nat Med.

[16] Nakahara J, Kanekura K, Nawa M, Aiso S, Suzuki N (2009). Abnormal expression of TIP30 and arrested nucleocytoplasmic transport within oligodendrocyte precursor cells in multiple sclerosis. J Clin Invest.

[17] Zavadil J, Cermak L, Soto-Nieves N, Bottinger EP (2004). Integration of TGF-beta/Smad and Jagged1/Notch signalling in epithelial-to-mesenchymal transition. EMBO J.

[18] Hung YH, Hung WC (2009). 4-(Methylnitrosamino)-1-(3-pyridyl)-1-butanone (NNK) enhances invasiveness of lung cancer cells by up-regulating contactin-1 via the alpha7 nicotinic acetylcholine receptor/ERK signaling pathway. Chem Biol Interact.

[19] Hoek KS, Schlegel NC, Eichhoff OM, Widmer DS, Praetorius C, Einarsson SO, Valgeirsdottir S, Bergsteinsdottir K, Schepsky A, Dummer R, Steingrimsson E (2008). Novel MITF targets identified using a two-step DNA microarray strategy. Pigment Cell Melanoma Res.

[20] Wu M, Hemesath TJ, Takemoto CM, Horstmann MA, Wells AG, Price ER, Fisher DZ, Fisher DE (2000). c-Kit triggers dual phosphorylations, which couple activation and degradation of the essential melanocyte factor Mi. Genes Dev.

[21] Graves LM, Guy HI, Kozlowski P, Huang M, Lazarowski E, Pope RM, Collins MA, Dahlstrand EN, Earp HS, Evans DR (2000). Regulation of carbamoyl phosphate synthetase by MAP kinase. Nature.

[22] Wostradowski T, Prajeeth CK, Gudi V, Kronenberg J, Witte S, Brieskorn M, Stangel M (2016). In vitro evaluation of physiologically relevant concentrations of teriflunomide on activation and proliferation of primary rodent microglia. J Neuroinflammation.

[23] Diedrichs-Mohring M, Leban J, Strobl S, Obermayr F, Wildner G (2014). A new small molecule for treating inflammation and chorioretinal neovascularization in relapsing-remitting and chronic experimental autoimmune uveitis. Invest Ophthalmol Vis Sci.

[24] Duan W, Chan JH, Wong CH, Leung BP, Wong WS (2004). Anti-inflammatory effects of mitogen-activated protein kinase kinase inhibitor U0126 in an asthma mouse model. J Immunol.

[25] Peterson JW, Bo L, Mork S, Chang A, Ransohoff RM, Trapp BD (2002). VCAM-1-positive microglia target oligodendrocytes at the border of multiple sclerosis lesions. J Neuropathol Exp Neurol.

[26] Polman CH, O'Connor PW, Havrdova E, Hutchinson M, Kappos L, Miller DH, Phillips JT, Lublin FD, Giovannoni G, Wajgt A, Toal M, Lynn F, Panzara MA, Sandrock AW (2006). A randomized, placebo-controlled trial of natalizumab for relapsing multiple sclerosis. N Engl J Med.

[27] Chen Y, Xie HQ, Sha R, Xu T, Zhang S, Fu H, Xia Y, Liu Y, Xu L, Zhao B (2020). 2,3,7,8-Tetrachlorodibenzo-p-dioxin and up-regulation of neurofilament expression in neuronal cells: Evaluation of AhR and MAPK pathways. Environ Int.

[28] Thebault S, Abdoli M, Fereshtehnejad SM, Tessier D, Tabard-Cossa V, Freedman MS (2020). Serum neurofilament light chain predicts long term clinical outcomes in multiple sclerosis. Sci Rep.

[29] Kassubek R, Gorges M, Schocke M, Hagenston VAM, Huss A, Ludolph AC, Kassubek J, Tumani H (2017). GFAP in early multiple sclerosis: a biomarker for inflammation. Neurosci Lett.

[30] Housley WJ, Pitt D, Hafler DA (2015). Biomarkers in multiple sclerosis. Clin Immunol.

[31] Sticozzi C, Belmonte G, Meini A, Carbotti P, Grasso G, Palmi M (2013). IL-1beta induces GFAP expression in vitro and in vivo and protects neurons from traumatic injury-associated apoptosis in rat brain striatum via NFkappaB/Ca(2)(+)-calmodulin/ERK mitogen-activated protein kinase signaling pathway. Neuroscience.

[32] Li D, Tong L, Kawano H, Liu N, Yan HJ, Zhao L, Li HP (2016). Regulation and role of ERK phosphorylation in glial cells following a nigrostriatal pathway injury. Brain Res.

[33] Alexander JS, Harris MK, Wells SR, Mills G, Chalamidas K, Ganta VC, McGee J, Jennings MH, Gonzalez-Toledo E, Minagar A (2010). Alterations in serum MMP-8, MMP-9, IL-12p40 and IL-23 in multiple sclerosis patients treated with interferon-beta1b. Mult Scler.

[34] Fainardi E, Castellazzi M, Bellini T, Manfrinato MC, Baldi E, Casetta I, Paolino E, Granieri E, Dallocchio F (2006). Cerebrospinal fluid and serum levels and intrathecal production of active matrix metalloproteinase-9 (MMP-9) as markers of disease activity in patients with multiple sclerosis. Mult Scler.

[35] Iyer V, Pumiglia K, DiPersio CM (2005). Alpha3beta1 integrin regulates MMP-9 mRNA stability in immortalized keratinocytes: a novel mechanism of integrin-mediated MMP gene expression. J Cell Sci.

[36] Munoz-San Martin M, Reverter G, Robles-Cedeno R, Buxo M, Ortega FJ, Gomez I, Tomas-Roig J, Celarain N, Villar LM, Perkal H (2019). Analysis of miRNA signatures in CSF identifies upregulation of miR-21 and miR-146a/b in patients with multiple sclerosis and active lesions. J Neuroinflammation.

[37] Mei Y, Bian C, Li J, Du Z, Zhou H, Yang Z, Zhao RC (2013). miR-21 modulates the ERK-MAPK signaling pathway by regulating SPRY2 expression during human mesenchymal stem cell differentiation. J Cell Biochem.

[38] Shukla A, Rai K, Shukla V, Chaturvedi NK, Bociek RG, Pirruccello SJ, Band H, Lu R, Joshi SS (2016). Sprouty 2: a novel attenuator of B-cell receptor and MAPK-Erk signaling in CLL. Blood.

[39] Liu S, Rezende RM, Moreira TG, Tankou SK, Cox LM, Wu M, Song A, Dhang FH, Wei Z, Costamagna G, Weiner HL (2019). Oral administration of miR-30d from feces of MS patients suppresses MS-like symptoms in mice by expanding Akkermansia muciniphila. Cell Host Microbe.

[40] Ye C, Yu X, Liu X, Dai M, Zhang B (2018). miR-30d inhibits cell biological progression of Ewing’s sarcoma by suppressing the MEK/ERK and PI3K/Akt pathways in vitro. Oncol Lett.

[41] Zhu QY, Liu Q, Chen JX, Lan K, Ge BX (2010). MicroRNA-101 targets MAPK phosphatase-1 to regulate the activation of MAPKs in macrophages. J Immunol.

[42] Caunt CJ, Rivers CA, Conway-Campbell BL, Norman MR, McArdle CA (2008). Epidermal growth factor receptor and protein kinase C signaling to ERK2: spatiotemporal regulation of ERK2 by dual specificity phosphatases. J Biol Chem.

[43] Wu T, Chen G (2016). miRNAs participate in MS pathological processes and its therapeutic response. Mediat Inflamm.

[44] Chen J, Zhu J, Wang Z, Yao X, Wu X, Liu F, Zheng W, Li Z, Lin A (2017) MicroRNAs correlate with multiple sclerosis and neuromyelitis optica spectrum disorder in a Chinese population. Med Sci Monit 23: 2565-2583. DOI 10.12659/msm.904642 10.12659/MSM.904642 PMC5458669 28550707

[45] Ahmad MK, Abdollah NA, Shafie NH, Yusof NM, Razak SRA (2018). Dual-specificity phosphatase 6 (DUSP6): a review of its molecular characteristics and clinical relevance in cancer. Cancer Biol Med.

[46] Zhang Z, Kobayashi S, Borczuk AC, Leidner RS, Laframboise T, Levine AD, Halmos B (2010). Dual specificity phosphatase 6 (DUSP6) is an ETS-regulated negative feedback mediator of oncogenic ERK signaling in lung cancer cells. Carcinogenesis.

[47] Wosik K, Antel J, Kuhlmann T, Bruck W, Massie B, Nalbantoglu J (2003). Oligodendrocyte injury in multiple sclerosis: a role for p53. J Neurochem.

[48] Suzuki HI, Yamagata K, Sugimoto K, Iwamoto T, Kato S, Miyazono K (2009). Modulation of microRNA processing by p53. Nature.

[49] Sachdeva M, Zhu S, Wu F, Wu H, Walia V, Kumar S, Elble R, Watabe K, Mo YY (2009). p53 represses c-Myc through induction of the tumor suppressor miR-145. Proc Natl Acad Sci U S A.

[50] Lee JC, Laydon JT, McDonnell PC, Gallagher TF, Kumar S, Green D, McNulty D, Blumenthal MJ, Heys JR, Landvatter SW (1994). A protein kinase involved in the regulation of inflammatory cytokine biosynthesis. Nature.

[51] Nick JA, Young SK, Arndt PG, Lieber JG, Suratt BT, Poch KR, Avdi NJ, Malcolm KC, Taube C, Henson PM, Worthen GS (2002). Selective suppression of neutrophil accumulation in ongoing pulmonary inflammation by systemic inhibition of p38 mitogen-activated protein kinase. J Immunol.

[52] Sondergaard HB, Hesse D, Krakauer M, Sorensen PS, Sellebjerg F (2013). Differential microRNA expression in blood in multiple sclerosis. Mult Scler.

[53] Perry MM, Williams AE, Tsitsiou E, Larner-Svensson HM, Lindsay MA (2009). Divergent intracellular pathways regulate interleukin-1beta-induced miR-146a and miR-146b expression and chemokine release in human alveolar epithelial cells. FEBS Lett.

[54] Cameron JE, Yin Q, Fewell C, Lacey M, McBride J, Wang X, Lin Z, Schaefer BC, Flemington EK (2008). Epstein-Barr virus latent membrane protein 1 induces cellular MicroRNA miR-146a, a modulator of lymphocyte signaling pathways. J Virol.

[55] Junker A, Krumbholz M, Eisele S, Mohan H, Augstein F, Bittner R, Lassmann H, Wekerle H, Hohlfeld R, Meinl E (2009). MicroRNA profiling of multiple sclerosis lesions identifies modulators of the regulatory protein CD47. Brain.

[56] Masliah-Planchon J, Garinet S, Pasmant E (2016). RAS-MAPK pathway epigenetic activation in cancer: miRNAs in action. Oncotarget.

[57] Freiesleben S, Hecker M, Zettl UK, Fuellen G, Taher L (2016). Analysis of microRNA and gene expression profiles in multiple sclerosis: integrating interaction data to uncover regulatory mechanisms. Sci Rep.

[58] Lightell DJ, Moss SC, Woods TC (2018). Upregulation of miR-221 and -222 in response to increased extracellular signal-regulated kinases 1/2 activity exacerbates neointimal hyperplasia in diabetes mellitus. Atherosclerosis.

[59] Ma XL, Shang F, Ni W, Zhu J, Luo B, Zhang YQ (2018). MicroRNA-338-5p plays a tumor suppressor role in glioma through inhibition of the MAPK-signaling pathway by binding to FOXD1. J Cancer Res Clin Oncol.

[60] De Santis G, Ferracin M, Biondani A, Caniatti L, Rosaria Tola M, Castellazzi M, Zagatti B, Battistini L, Borsellino G, Fainardi E (2010). Altered miRNA expression in T regulatory cells in course of multiple sclerosis. J Neuroimmunol.

[61] Bayraktar R, Van Roosbroeck K, Calin GA (2017). Cell-to-cell communication: microRNAs as hormones. Mol Oncol.

[62] Mutlu M, Saatci O, Ansari SA, Yurdusev E, Shehwana H, Konu O, Raza U, Sahin O (2016). miR-564 acts as a dual inhibitor of PI3K and MAPK signaling networks and inhibits proliferation and invasion in breast cancer. Sci Rep.

[63] Pang Y, Zheng B, Kimberly SL, Cai Z, Rhodes PG, Lin RC (2012). Neuron-oligodendrocyte myelination co-culture derived from embryonic rat spinal cord and cerebral cortex. Brain Behav.

[64] Li J, Ramenaden ER, Peng J, Koito H, Volpe JJ, Rosenberg PA (2008). Tumor necrosis factor alpha mediates lipopolysaccharide-induced microglial toxicity to developing oligodendrocytes when astrocytes are present. J Neurosci.

[65] Li DQ, Luo L, Chen Z, Kim HS, Song XJ, Pflugfelder SC (2006). JNK and ERK MAP kinases mediate induction of IL-1beta, TNF-alpha and IL-8 following hyperosmolar stress in human limbal epithelial cells. Exp Eye Res.

[66] Kaur C, Rathnasamy G, Ling EA (2013). Roles of activated microglia in hypoxia induced neuroinflammation in the developing brain and the retina. J NeuroImmune Pharmacol.

[67] Nave KA, Trapp BD (2008). Axon-glial signaling and the glial support of axon function. Annu Rev Neurosci.

[68] Funfschilling U, Supplie LM, Mahad D, Boretius S, Saab AS, Edgar J, Brinkmann BG, Kassmann CM, Tzvetanova ID, Mobius W (2012). Glycolytic oligodendrocytes maintain myelin and long-term axonal integrity. Nature.

[69] Benveniste EN (1997). Role of macrophages/microglia in multiple sclerosis and experimental allergic encephalomyelitis. J Mol Med (Berl).

[70] Lublin FD, Coetzee T, Cohen JA, Marrie RA, Thompson AJ, International Advisory Committee on Clinical Trials in MS (2020). The 2013 clinical course descriptors for multiple sclerosis: a clarification. Neurology.

[71] Lublin FD, Reingold SC, Cohen JA, Cutter GR, Sorensen PS, Thompson AJ, Wolinsky JS, Balcer LJ, Banwell B, Barkhof F, Bebo B, Calabresi PA, Clanet M, Comi G, Fox RJ, Freedman MS, Goodman AD, Inglese M, Kappos L, Kieseier BC, Lincoln JA, Lubetzki C, Miller AE, Montalban X, O'Connor PW, Petkau J, Pozzilli C, Rudick RA, Sormani MP, Stuve O, Waubant E, Polman CH (2014). Defining the clinical course of multiple sclerosis: the 2013 revisions. Neurology.

[72] ‘t Hart BA, Luchicchi A, Schenk GJ, Geurts JJG (2021) Mechanistic underpinning of an inside-out concept for autoimmunity in multiple sclerosis. submitted for publication 10.1002/acn3.51401 PMC8351380 34156169

[73] Luchicchi A, Hart B, Frigerio I, van Dam AM, Perna L, Offerhaus HL, Stys PK, Schenk GJ, Geurts JJG (2021). Axon-myelin unit blistering as early event in ms normal appearing white matter. Ann Neurol.

[74] Singh S, Metz I, Amor S, van der Valk P, Stadelmann C, Bruck W (2013). Microglial nodules in early multiple sclerosis white matter are associated with degenerating axons. Acta Neuropathol.

[75] Reyskens KM, Arthur JS (2016). Emerging roles of the mitogen and stress activated kinases MSK1 and MSK2. Front Cell Dev Biol.

[76] Caunt CJ, Keyse SM (2013). Dual-specificity MAP kinase phosphatases (MKPs): shaping the outcome of MAP kinase signalling. FEBS J.

[77] Keller A, Leidinger P, Lange J, Borries A, Schroers H, Scheffler M, Lenhof HP, Ruprecht K, Meese E (2009). Multiple sclerosis: microRNA expression profiles accurately differentiate patients with relapsing-remitting disease from healthy controls. PLoS ONE.

[78] Ma X, Zhou J, Zhong Y, Jiang L, Mu P, Li Y, Singh N, Nagarkatti M, Nagarkatti P (2014). Expression, regulation and function of microRNAs in multiple sclerosis. Int J Med Sci.

[79] Gu Y, Li D, Luo Q, Wei C, Song H, Hua K, Song J, Luo Y, Li X, Fang L (2015). MicroRNA-145 inhibits human papillary cancer TPC1 cell proliferation by targeting DUSP6. Int J Clin Exp Med.

[80] Zhang Y, Leung DY, Richers BN, Liu Y, Remigio LK, Riches DW, Goleva E (2012). Vitamin D inhibits monocyte/macrophage proinflammatory cytokine production by targeting MAPK phosphatase-1. J Immunol.

[81] Franklin CC, Kraft AS (1997). Conditional expression of the mitogen-activated protein kinase (MAPK) phosphatase MKP-1 preferentially inhibits p38 MAPK and stress-activated protein kinase in U937 cells. J Biol Chem.

[82] Taylor DM, Moser R, Regulier E, Breuillaud L, Dixon M, Beesen AA, Elliston L, Silva Santos Mde F, Kim J, Jones L (2013). MAP kinase phosphatase 1 (MKP-1/DUSP1) is neuroprotective in Huntington's disease via additive effects of JNK and p38 inhibition. J Neurosci.

[83] Sanchez-Tillo E, Comalada M, Xaus J, Farrera C, Valledor AF, Caelles C, Lloberas J, Celada A (2007). JNK1 Is required for the induction of Mkp1 expression in macrophages during proliferation and lipopolysaccharide-dependent activation. J Biol Chem.

[84] Steinmetz R, Wagoner HA, Zeng P, Hammond JR, Hannon TS, Meyers JL, Pescovitz OH (2004). Mechanisms regulating the constitutive activation of the extracellular signal-regulated kinase (ERK) signaling pathway in ovarian cancer and the effect of ribonucleic acid interference for ERK1/2 on cancer cell proliferation. Mol Endocrinol (Baltimore, Md).

[85] Tomida T, Takekawa M, Saito H (2015). Oscillation of p38 activity controls efficient pro-inflammatory gene expression. Nat Commun.

[86] Smolders J, Torkildsen O, Camu W, Holmoy T (2019). An update on vitamin D and disease activity in multiple sclerosis. CNS Drugs.

[87] Roberts ML, Cooper NR (1998). Activation of a ras-MAPK-dependent pathway by Epstein-Barr virus latent membrane protein 1 is essential for cellular transformation. Virology.

[88] Rosato P, Anastasiadou E, Garg N, Lenze D, Boccellato F, Vincenti S, Severa M, Coccia EM, Bigi R, Cirone M, Ferretti E, Campese AF, Hummel M, Frati L, Presutti C, Faggioni A, Trivedi P (2012). Differential regulation of miR-21 and miR-146a by Epstein-Barr virus-encoded EBNA2. Leukemia.

[89] Hassani A, Corboy JR, Al-Salam S, Khan G (2018). Epstein-Barr virus is present in the brain of most cases of multiple sclerosis and may engage more than just B cells. PLoS ONE.

[90] Lin KM, Lin SJ, Lin JH, Lin PY, Teng PL, Wu HE, Yeh TH, Wang YP, Chen MR, Tsai CH (2020) Dysregulation of dual-specificity phosphatases by Epstein-Barr virus LMP1 and its impact on lymphoblastoid cell line survival. J Virol 94. 10.1128/JVI.01837-19 10.1128/JVI.01837-19 PMC6997745 31776277

[91] Oaks JJ, Santhanam R, Walker CJ, Roof S, Harb JG, Ferenchak G, Eisfeld AK, Van Brocklyn JR, Briesewitz R, Saddoughi SA (2013). Antagonistic activities of the immunomodulator and PP2A-activating drug FTY720 (Fingolimod, Gilenya) in Jak2-driven hematologic malignancies. Blood.

[92] Shrestha J, Ki SH, Shin SM, Kim SW, Lee JY, Jun HS, Lee T, Kim S, Baek DJ, Park EY (2018) Synthesis of novel FTY720 analogs with anticancer activity through PP2A activation. Molecules 23. 10.3390/molecules23112750 10.3390/molecules23112750 PMC6278267 30355990

[93] Tai AK, O'Reilly EJ, Alroy KA, Simon KC, Munger KL, Huber BT, Ascherio A (2008). Human endogenous retrovirus-K18 Env as a risk factor in multiple sclerosis. Mult Scler.

[94] Lemaitre C, Tsang J, Bireau C, Heidmann T, Dewannieux M (2017). A human endogenous retrovirus-derived gene that can contribute to oncogenesis by activating the ERK pathway and inducing migration and invasion. PLoS Pathog.

[95] Kim SH, Smith CJ, Van Eldik LJ (2004). Importance of MAPK pathways for microglial pro-inflammatory cytokine IL-1 beta production. Neurobiol Aging.

[96] Troscher AR, Wimmer I, Quemada-Garrido L, Kock U, Gessl D, Verberk SGS, Martin B, Lassmann H, Bien CG, Bauer J (2019). Microglial nodules provide the environment for pathogenic T cells in human encephalitis. Acta Neuropathol.

[97] Mei YP, Zhou JM, Wang Y, Huang H, Deng R, Feng GK, Zeng YX, Zhu XF (2007). Silencing of LMP1 induces cell cycle arrest and enhances chemosensitivity through inhibition of AKT signaling pathway in EBV-positive nasopharyngeal carcinoma cells. Cell Cycle.

[98] Wang J, Quake SR (2014). RNA-guided endonuclease provides a therapeutic strategy to cure latent herpesviridae infection. Proc Natl Acad Sci U S A.

